# A fatal and unusual genital mutilation in an elderly man as a result of sharp force injuries and domestic dog predation

**DOI:** 10.1007/s12024-022-00542-w

**Published:** 2022-10-14

**Authors:** Francesco Simonit, Carlo Moreschi, Lorenzo Desinan

**Affiliations:** Dipartimento di Area Medica, Università degli Studi di Udine, Medicina Legale, piazzale Santa Maria della Misericordia 15, 33100 Udine, Italy

**Keywords:** Genital mutilation, Animal predation, Dog attack, Self-harm, Forensic, Autopsy

## Abstract

Cases of genital amputation require a careful investigation, since they may be the result of self-inflicted injuries, assaults, animal predation, or post-mortem body mutilation. In the present case, an 81-year-old man affected by liver cirrhosis and dementia and suffering from sexual disinhibition was found lying half-naked and unconscious in his courtyard; profuse bleeding in the perineal area and the absence of the external genitalia were observed. The victim was transferred to hospital and underwent surgical emergency treatment, but he died 2 days later. No sharp tools were found on the scene. Moreover, the autopsy did not reveal any defense or tentative wound on the body and no blood stains on the hands of the victim. Several linear scratches were detected close to the edges of the wound; according to the surgical report, these scratches were clean-cut in the lower part and crenated and infiltrated by blood in the cranial part. Although the medical history of the man could be consistent with self-mutilation, it was not possible to rule out the involvement of other people, including the possibility of an attempt by his relatives to cover up what may have been a self-amputation. Furthermore, the victim’s dog vomited parts of the man’s genitalia while being transported to a dog shelter. Similar cases have rarely been published in the current forensic literature.

## Introduction

Male genital mutilations include a wide spectrum of lesions, ranging from cultural practices and the insertion of plastic spherules under the skin of the penis to the removal of one or both testes (castration) or the complete amputation of penis, scrotum and testes. They may be self-inflicted or arise from accidents, assaults and animal predation, and may occur before, after or around the time of death [1–3].

The aim of this paper is to present an unusual case involving an elderly man affected by dementia who was found bleeding due to a complete mutilation of the genitals. He underwent reconstructive surgery, but died two days later. The man’s dog vomited part of his genitalia. The injury in the pubic area was partially characterized by clean edges, but no weapon was found during the scene investigation.

The case occurred in 2001 and was mentioned in a previous study [4].

## Case report

One day in September around 11.30 am, an 81-year-old man was found lying unconscious and supine in a pool of blood in his courtyard by his caregiver. The man was wearing an undershirt, which was smeared with blood in its lower part, and was bleeding from a wound in the genital region. The caregiver called the paramedics and some of the man’s relatives, and the victim was transferred to hospital.

Several relatives of the victim were on the scene at the arrival of the investigators. No other blood patterns were noted. Furthermore, there were no signs of struggle or burglary and no sharp tools were found.

According to the paramedics, blood was only present in the genital area and on the lower abdominal regions of the victim; no blood was found on his hands and forearms.

**Fig. 1:**
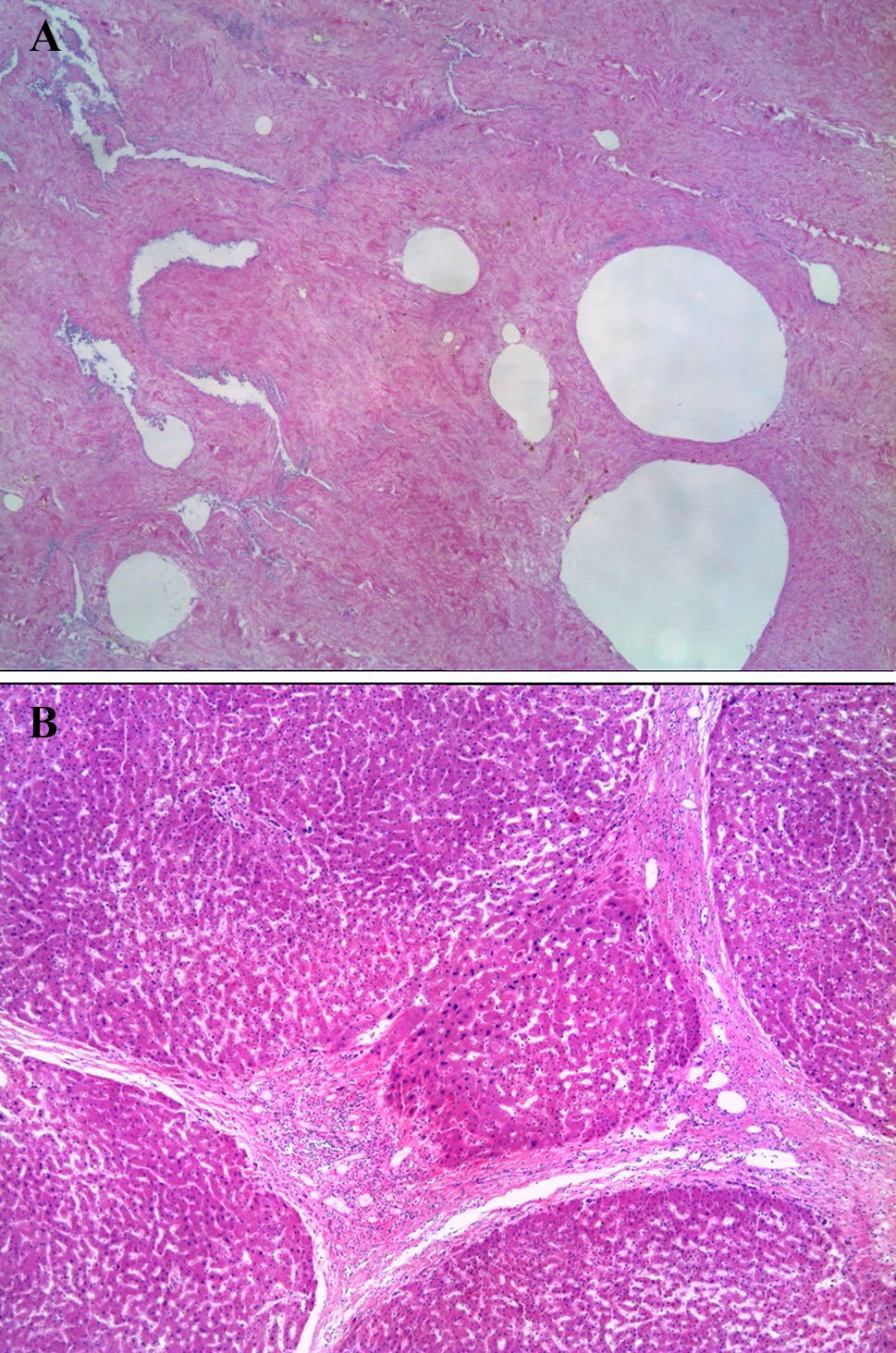
**A**, **B** Histological aspect of the material vomited by the dog (penile tissue with cavernous sinuses) and of the liver of the victim (hematoxylin–eosin, 50×)

The victim had a small half-breed dog, male and unneutered, which vomited while being carried to a dog shelter. The vomited material was found to contain part of the victim’s genitalia, as confirmed by the histological examination (Fig. 1A). The prosecutor did not order to put down the pet.

On arrival at the emergency department, GCS was 8/15, blood pressure was 60/40 mmHg and Hb levels were 10.2 g/dL. Profuse bleeding was noted in the genital area, and head CT scans were negative for acute lesions. The man was intubated and brought to the operating room.

According to the surgeon’s report, the lower edges of the wound were sharp, while the upper ones were irregular, crenated and showed a subcutaneous hemorrhage.

Wound exploration and debridement were performed before oversewing the corpora cavernosa. What was left of the bulbous urethra was re-fashioned through a perineal urethrostomy, and a urethral catheter was inserted. Despite being transferred to the intensive care unit (ICU) and being treated with blood transfusions, packed red blood cells, oxygen supply, and positive inotropic agents, the man died two days after admission.

The man suffered from sexual disinhibition caused by dementia, had been treated for a left temporal intracerebral hemorrhage, and had a history of alcohol abuse. Furthermore, he suffered from persecutory delusions and used to hide a large knife (which was not recovered) under his pillow.

**Fig. 2:**
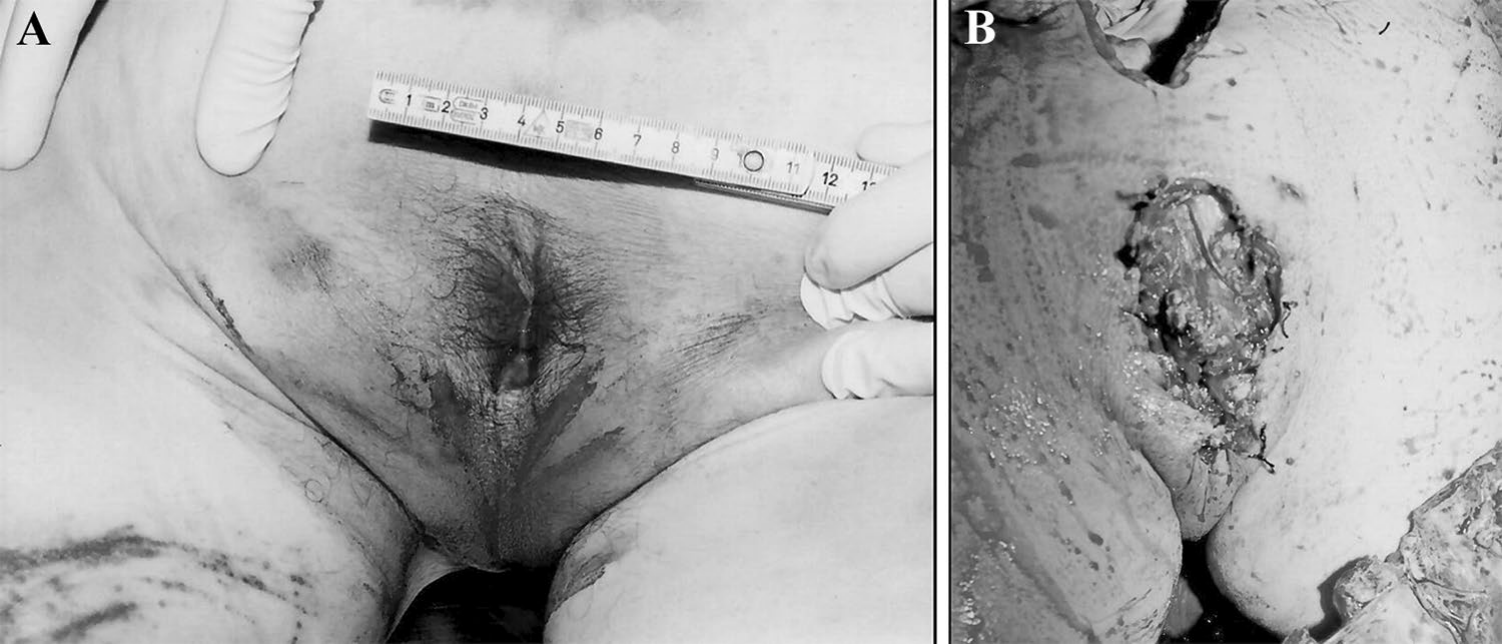
**A**, **B** The surgical wound (length = 10 cm) in the genital area during the external examination and after the exploration. Clustered abrasions can be noted on the right side of the groin

At autopsy (performed one day later), different clusters of parallel abrasions were noted on the right trochanteric region, on the right side of the groin and on the medial aspect of the left thigh. No injuries were observed on the upper limbs. A longitudinal surgical incision sewn with staples was found in the pubic region. Blood-infiltration of the edges of the wound was slight in the lower part of the injury and more significant in the upper part (Fig. 2).

Other relevant findings included cerebral atrophy, liver cirrhosis (Fig. 1B), coronary atherosclerosis, myocardial sclerosis, and pressure lesions on the sacral region.

Toxicology was performed on peripheral blood that had been sampled on admission to hospital before blood transfusion, on bile specimens collected at autopsy and on the gastric content of the dog. Traces of nicotine and lidocaine were found in the blood samples. The cause of death was attributed to hypoxic ischemic encephalopathy resulting from exsanguination caused by a mutilation to the genital area.

## Discussion

Autopsies on bodies with genital mutilation require a careful approach in order to correctly establish the manner of death [3, 5]. In the case here discussed, several peculiar traits had to be considered.

### The operative management of the injury

Death due to hemorrhage as a result of genital mutilations is rare [2, 6, 7]. In the present case, the severity of the injury, the age of the victim and the cirrhosis from which he suffered are likely to have contributed to the fatal outcome, despite the fluid resuscitation, the respiratory support and the lack of complications during the surgical procedure [6, 8, 9].

### Analysis of the injury patterns

At autopsy, the aforementioned surgical treatment influenced the analysis of the wound. In such cases, the medical record can provide crucial information, since surgery and hospitalization may result in the alteration or onset of wounds [10].

The vomited material included part of the victim’s genitalia. During the examination of the wound in the operating room, the edges were found to be cut-off in the lower part, while they were irregular, crenated and accompanied by scratch-like abrasions in the upper part.

The margins of the lower part were consistent with a sharp-force injury, whereas, as regards the upper margins and the vomited genitalia, the hypothesis of dog predation had to be considered.

Wounds inflicted by dogs can be classified as non-fatal bite wounds, fatal bite wounds, and post-mortem lacerations [11].

Dogs usually target the head, the neck, the throat and any exposed part of the body, with the genitalia being occasionally consumed [11–13]. Dogs immobilize their victims by striking their limbs, and attack the upper half of the victim’s body after he/she has been brought down [14].

The typical injury pattern caused by dog bites, named “hole and tear”, is the consequence of a combination of biting, clawing, and crushing, with a round “hole” made by the canine tooth penetrating into the skin, whereas the other teeth cut into the flesh and cause stretch lacerations due to the shaking of the head (the “tear”) [11, 12, 14].

Considering the lack of bite wounds on the lower limbs and the absence of defense injuries, it was likely that the man was already lying on the ground when the dog attacked him and that he did not attempt to defend himself.

Furthermore, taking into consideration the features of the wound and the presence of parallel scratches close to the injury, the hypothesis of the dog having amputated and then consumed the genitals could be ruled out.

Since the interpretation of dog-inflicted wounds can be misleading, an odontological examination of the bite marks and the DNA analysis of the saliva recovered on the victim’s body can assist the forensic pathologist in identifying the animal [15, 16].

In the present case, such methods might have been affected by the way the wound had been treated during surgery.

The peculiarity of this case consists in the fact that the victim was still alive when his genitals were eaten: cases of dogs eating their dead owners have been frequently reported, whereas dog injuries are extremely rare on unconscious victims [17–19].

The literature reports the case of a dog that vomited human parts and dog food, as well as cases of post-mortem genital predation by dogs on a 49-year-male who had died of myocardial infarction and on a young man who had hanged himself [3, 13, 20].

Another key point was the presence of sharp edges in the lower part of the wound, which raised the possibility of an assault.

Given that no signs of struggle were noticed and that blood was only found on the man’s body, the fact may have occurred in the same place where the man was found.

However, the involvement of other people could not be ruled out, as the scene may have been altered by the victim’s relatives in order to cover up the nature of the self-injury [21, 22].

The lack of damage to the undershirt and the presence of the injury on an unclothed part of the body were consistent with a case of self-injury, but could not rule out an aggression. Tentative wounds were not observed [23].

Moreover, defense wounds may also be missing in homicide victims who suffer a single sharp-force injury, if the victim is attacked suddenly or if his/her ability to act is reduced. The latter hypothesis was not supported by the toxicological and autopsy findings [24–26].

Homicidal injuries to the genital area are rare and are typically associated with sadistic and sexual homicides [3, 27]. Perpetrators may be affected by anti-social disorders or paraphilias and tend to be men, whereas victims tend to be adult women.

Cases of elderly victims (mostly women) often involve other offenses, such as burglary [28].

A considerable number of non-fatal cases where women cut off the penis of their male partner while the man slept was documented in Thailand in the 1970s [29].

In the present case, since the victim was found in his courtyard and no signs of struggle were found, the abovementioned hypothesis was unlikely.

The other hypothesis concerned male genital self-mutilation (GSM) resulting in complete emasculation [2, 8].

Suicidal attempts through GSM are rare and often unsuccessful, while GSM is more frequent in cases of self-harm [2, 7, 30–32].

GSM is often associated with psychiatric conditions. However, GSM is sometimes performed by non-psychotic individuals for aesthetic and social purposes, for distorted religious beliefs or to seek sexual reassignment surgery [1, 33–38].

The large majority of individuals performing GSM are males in the third and fourth decades of life [1, 39], with the oldest victims being aged 62, 66, and 72 [35, 40–42].

In a recent paper, a 60-year-old man with a history of fronto-temporal dementia inserted a spoon into the urethra during a stay in hospital [43].

## Concluding remarks

In the present case, a complete amputation of the genitals was observed in an elderly man who suffered from dementia and sexual disinhibition and who was found lying unconscious and bleeding in his courtyard. Despite reconstructive surgery, he died two days later. The dog of the victim vomited part of the man’s genitalia.

According to the surgeon’s report, the wound had sharp edges on its lower part and irregular and crenated edges with subcutaneous hemorrhage on the upper part, leading to the hypothesis that the amputation may have been the result of a cut wound, followed by ante-mortem dog predation with the victim lying on the ground. However, although the absence of defense injuries and the history of the victim were suggestive of self-mutilation, the lack of a weapon could not rule out the involvement of a third party.

Consequently, the death remained undetermined, since, according to Italian law, the manner of death must be ascertained as per the legal standard of “beyond reasonable doubt”.

This case was characterized by several key points.

Genital amputation, whether self-inflicted or performed by assailants, is uncommon among elderly, with fatal cases being rare.

Furthermore, sharp-force injuries are rarely associated with ante-mortem dog predation.

Lastly, if a victim has been hospitalized and undergone surgery before dying, the importance of the medical records should not be understated, since injuries may be altered considerably during surgery.

## Key points

- An elderly man was found unconscious in his courtyard without external genitalia
- No sharp tools were found and the victim’s dog vomited part of his genitalia
- Tentative and defense wounds were not present and the man died after surgery
- The lower wound edges were sharp, while the upper ones were infiltrated by blood
- Neither self-mutilation nor the involvement of other people could be ruled out

## References

[1] Veeder TA, Leo RJ (2017). Male genital self-mutilation: a systematic review of psychiatric disorders and psychosocial factors. Gen Hosp Psychiatry.

[2] Murphy D, Murphy M, Grainger R (1995). Self-castration. Ir J Med Sci.

[3] Byard RW (2017). Implications of genital mutilation at autopsy. J Forensic Sci.

[4] Mazzolo GM, Desinan L (2005). Sharp force fatalities: suicide, homicide or accident? A series of 21 cases. Forensic Sci Int.

[5] Dobiáš M, Marecová K, Vránová K, Handlos P (2019). Penile carcinoma – a rare cause of sudden death. Forensic Sci Med Pathol.

[6] Stunell H, Power RE, Floyd M, Quinlan DM (2006). Genital self-mutilation. Int J Urol.

[7] Romilly CS, Isaac MT (1996). Male genital self-mutilation. Br J Hosp Med.

[8] Wilcox Vanden Berg RN, Gaffney CD, Paduch DA (2020). Presentation and resolution of gender dysphoria as a positive symptom in a young schizophrenic man who presented with self-emasculation: Frontiers of bioethics, psychiatry, and microsurgical genital reconstruction. Clin Case Reports..

[9] Koller CR, Wang S, Sandoval V, Yousif A, Hsieh TC, Raheem OA. Self-induced trauma to the genitalia: a review of the literature and management schemes. Curr Urol Rep. 2021;22. 10.1007/s11934-021-01034-0 33534050

[10] Shkrum MJ, Ramsay DA, Shkrum MJ (2007). Penetrating trauma. Forensic Pathol Trauma Common Probl forensic Pathol.

[11] Santoro V, Smaldone G, Lozito P, Smaldone M, Introna F (2011). A forensic approach to fatal dog attacks. A case study and review of the literature. Forensic Sci Int.

[12] Fonseca GM, Palacios R. An unusual case of predation: dog pack or cougar attack? J Forensic Sci. 2013: 224–7. 10.1111/j.1556-4029.2012.02281.x 22971181

[13] Romain N, Brandt-Casadevall C, Dimo-Simonin N, Michaud K, Mangin P, Papilloud J (2002). 3. Post-mortem castration by a dog: a case report. Med Sci Law.

[14] Bury D, Langlois N, Byard RW (2012). Animal-related fatalities-part I: characteristic autopsy findings and variable causes of death associated with blunt and sharp trauma. J Forensic Sci.

[15] Salem NH, Belhadj M, Aissaoui A, Mesrati MA, Chadly A (2013). Multidisciplinary approach to fatal dog attacks: A forensic case study. J Forensic Leg Med.

[16] Iarussi F, Cipolloni L, Bertozzi G, Sasso L, Ferrara M, Salerno M (2020). Dog-bite-related attacks: A new forensic approach. Forensic Sci Int.

[17] Buschmann C, Solarino B, Püschel K, Czubaiko F, Heinze S, Tsokos M (2011). Post-mortem decapitation by domestic dogs: Three case reports and review of the literature. Forensic Sci Med Pathol.

[18] Steadman DW, Worne H (2007). Canine scavenging of human remains in an indoor setting. Forensic Sci Int.

[19] Verzeletti A, Cortellini V, Vassalini M (2010). Post-mortem injuries by a dog: a case report. J Forensic Leg Med.

[20] Rothschild MA, Schneider V (1997). On the temporal onset of postmortem animal scavenging “Motivation” of the animal. Forensic Sci Int.

[21] Nadesan K, Beng OB (2001). 2. Two cases of death due to plastic bag suffocation. Med Sci Law.

[22] Visentin S, Massaro L, Viel G, Cecchetto G, Montisci M (2019). Suicide identification during on-site inspection. Proposal and application of an interpretative method for death scene investigation. Forensic Sci Int.

[23] Simonit F, Bassan F, Scorretti C, Desinan L (2018). Complex suicides: A review of the literature with considerations on a single case of abdominal self stabbing and plastic bag suffocation. Forensic Sci Int.

[24] Hunt AC, Cowling RJ (1991). Murder by stabbing. Forensic Sci Int.

[25] Racette S, Kremer C, Desjarlais A, Sauvageau A (2008). Suicidal and homicidal sharp force injury: A 5-year retrospective comparative study of hesitation marks and defense wounds. Forensic Sci Med Pathol.

[26] Pollak S, Saukko PJ, Knupfer GC (2000). Defense wounds. Encycl Forensic Sci.

[27] Karlsson T (1998). Homicidal and suicidal sharp force fatalities in Stockholm, Sweden. Orientation of entrance wounds in stabs gives information in the classification. Forensic Sci Int.

[28] Karakasi MV, Vasilikos E, Voultsos P, Vlachaki A, Pavlidis P (2017). Sexual homicide: Brief review of the literature and case report involving rape, genital mutilation and human arson. J Forensic Leg Med.

[29] Bhanganada K, Chayavatana T, Pongnumkul C, Tonmukayakul A, Sakolsatayadorn P, Komaratat K, Wilde H (1983). Surgical management of an epidemic of penile amputations in Siam. Am J Surg.

[30] Mishra KK, Reddy S, Khairkar P (2014). Genital self-mutilation in a suicide attempt: A rare sequela of a hypochondriacal delusion of infection with HIV. Int J STD AIDS.

[31] Rudhran V, Thippeswamy H, Chaturvedi SK (2013). Phallicide: A case of auto penile amputation as a mode of suicide. Aust N Z J Psychiatry.

[32] Koops E, Püschel K (1990). Self-mutilation and autophagia. Arch Kriminol.

[33] Anand JS, Habrat B, Barwina M, Waldman W (2015). Repeated self-mutilation of testicles in the context of methamphetamine use - a case report and brief review of literature. J Forensic Leg Med.

[34] Aboseif S, Gomez R, McAninch JW (1993). Genital self-mutilation. J Urol.

[35] Haleem S, Griffin SJ, Banerjee GK (2007). Self-castration: a case report. Gd Rounds.

[36] Hendershot E, Stutson AC, Adair TW (2010). A case of extreme sexual self-mutilation. J Forensic Sci.

[37] Baltieri DA, de Andrade AG. Transsexual genital self-castration. Am J Forensic Med Pathol. 2005;26:268–70. 10.1097/01.paf.0000163829.70005.bc 16121084

[38] Gleeson MJ, Connolly J, Grainger R (1993). Self-castration as treatment for alopecia. Br J Urol.

[39] Eke N, Davies A (2000). Genital self-mutilation: there is no method in this madness. BJU Int.

[40] Mattoo SK, Niraula A, Somani A (2018). Penile self-amputation for suicidal attempt in an elderly depressed case. Asian J Psychiatr.

[41] Hemphill RE (1951). A case of genital self-mutilation. Br J Med Psychol.

[42] Tharoor H (2007). A case of genital self-mutilation in an elderly man. Prim Care Companion J Clin Psychiatry.

[43] Adamec I, Klepac N, Mubrin Z, Habek M (2013). Genital self-mutilation in a patient with frontotemporal dementia. Neurol Croat.

